# Interactions of Polar and Nonpolar Groups of Alcohols in Zeolite Pores

**DOI:** 10.1021/jacs.5c09340

**Published:** 2025-07-12

**Authors:** Ruixue Zhao, Sungmin Kim, Mal-Soon Lee, Benjamin A. Jackson, Fuli Deng, Xiaomai Chen, Cong Zhou, Konstantin Khivantsev, Yue Liu, Vassiliki-Alexandra Glezakou, Roger Rousseau, Johannes A. Lercher

**Affiliations:** † Department of Chemistry and Catalysis Research Center, 9184 Technical University of Munich, Lichtenbergstrasse 4, 85748 Garching, Germany; ‡ Institute for Integrated Catalysis, 6865 Pacific Northwest National Laboratory, P.O. Box 999, Richland, Washington 99352, United States; § Shanghai Key Laboratory of Green Chemistry and Chemical Processes, School of Chemistry and Molecular Engineering, 539566 East China Normal University, Shanghai 200062, PR China

## Abstract

Understanding the quantitative interactions among zeolite pore walls, Bro̷nsted acid sites, and molecules with both polar and nonpolar regions is essential for scoping out the potential of zeolites as sorbents and catalysts. Purely siliceous zeolites (**MFI** and **Beta** in the present study) are hydrophobic, whereas those containing aluminum are considered hydrophilic, preferentially adsorbing organic molecules even in aqueous environments. To characterize these interactions, we use primary alcohols of increasing molecular weight, quantifying their specific interactions in the confined pore space of the alkyl (CH_*x*_) and OH groups. Three types of interactions were identified: (i) alkyl CH_*x*_ groups interacting with the zeolite pore walls (approximately 10 kJ mol^–1^ per carbon), (ii) alcohol OH groups interacting with the pore walls (30–35 kJ mol^–1^), and (iii) alcohol OH groups interacting with Bro̷nsted acid sites (37 kJ mol^–1^). All three interactions were well mirrored by computational simulations. The contribution of the alkyl CH_*x*_ groups was inferred from the incremental increase in sorption enthalpy with increasing molecular weight; the interaction strength of the OH groups was determined by extrapolating the global adsorption enthalpy of the alcohols to a hypothetical OH group without an alkyl group. This value was identical to the adsorption enthalpy of water. The experiments demonstrated that only water has an adsorption enthalpy on zeolite pore walls lower than its condensation enthalpy (30–35 kJ mol^–1^ vs 45 kJ mol^–1^), limiting the concentration of water that can be adsorbed.

## Graphical abstract

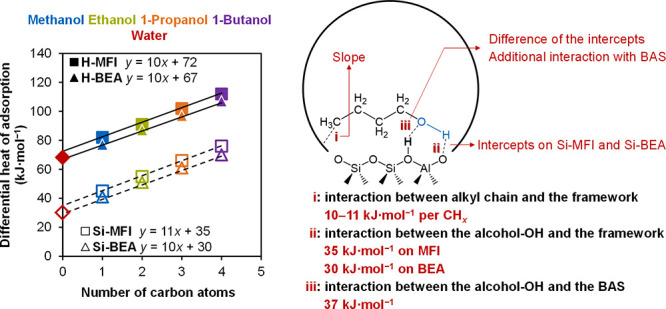

## Introduction

Zeolites are tectosilicates frequently used as catalysts and sorbents; their well-defined pores comprise the three-dimensionally linked silica (and alumina if substituted) tetrahedra that vary in polarity depending on their chemical composition.^1–4^ Colloquially, silica pore walls [Si­(OSi)_4_] are considered to be “hydrophobic”, while acid–base sites, such as bridging Si–OH–Al, are considered to be “hydrophilic”. The latter act as Bro̷nsted acid sites (BAS) and are considered catalytically active binding sites interacting with reactants, intermediates, and products.

Weak interactions of nonpolar molecules in these pores rely predominantly on nondirected dispersion forces (dynamic interactions caused by polarity or dipoles induced by correlated electron movements). With alkanes, for example, the strength of these interactions is primarily determined by the size of the specific channel and the pore structure. On the other hand, strong interactions arise from directed hydrogen bonding or Coulomb interactions between polar functional groups of sorbed molecules.^5,6^

The adsorption of nonpolar molecules, such as short-chain alkanes, within zeolite pores has been well-investigated over the past few decades. With dispersion forces dominating, adsorption is intricately governed by factors such as the size of the alkane, the zeolite pore structure, and the acid–base sites present.^6–20^ The differential heat of adsorption of alkanes increases with increasing alkane chain length, with each CH_*x*_ group contributing to the overall interaction: 10–12 kJ mol^–1^ for **MFI**,^7,21^ 9 kJ mol^–1^ for **MOR**,^22^ and 7 kJ mol^–1^ for **EMT** ^7^ and **FAU**.^7,22^ The presence of BAS or metal cations at zeolite exchange positions imparts a heteropolar nature, introducing permanent electrostatic interactions.^6,22–25^ We showed previously that the presence of BAS increases the adsorption enthalpy of alkanes by 10 kJ mol^–1^ for **MFI** and 6 kJ mol^–1^ for **FAU**. The difference has been tentatively attributed to differences in the acid strength.^7^

The adsorption of polar molecules such as water on zeolites containing BAS has been well-studied.^26–30^ For example, in H-MFI, the first adsorbed water molecule interacts with the BAS through hydrogen bonding, while the proton remains on the Si–OH–Al group,^26,30^ with an adsorption enthalpy of approximately 65 kJ mol^–1^.^28^ The heat of adsorption for the second water molecule (forming a dimer on BAS) increases to 85 kJ mol^–1^,^28^ attributed to the proton transfer from the BAS forming a hydrated hydronium ion,^30,31^ which has been characterized by high-resolution solid-state NMR spectroscopy.^30^ Additional water molecules (up to approximately 8 per BAS) stabilized this cluster and gradually decreased the enthalpy of adsorption to 45 kJ mol^–1^,^28^ comparable to the condensation enthalpy of water.^28,31^ As an ensemble, all adsorbed water molecules form hydrated hydronium ions, closely associated with the negative charge on the zeolite framework. The concentration of such hydrated hydronium ions influences the binding of other molecules. Increasing concentration reduces the adsorption enthalpy while increasing the excess chemical potential of the sorbate at the initial state.^32,33^ Hydronium ion concentration has been observed to cause a strong variation in the heat of sorption as well as an increase in the acid catalyzed reaction rates (e.g., alcohol dehydration) by lowering the excess chemical potential in the transition state; higher concentrations of hydronium ions lead to a decrease in the reaction rate due to rearrangement of the substrate and hydronium ion in the pore.^34,35^

As the alkyl group in alcohols is relatively nonpolar, while the hydroxyl group serves as a polar hydrogen-bonding site, the interactions between alcohols and zeolites encompass both dispersion forces and directed hydrogen bonding or protonation. Consequently, examining how alcohols interact within the pores of zeolites provides valuable insights into the distinct ways the silica framework and its discrete ion-exchange sites engage with the nonpolar and polar components of the sorbed molecules.

In the present work, we report the adsorption of short-chain (C_1_–C_4_) alcohols and water on **MFI** and **Beta** framework zeolites. In all samples, a linear correlation was observed between the heat of adsorption and the increasing carbon number, mirroring the enthalpy trends known from alkane adsorption.^7,21^ Calorimetry and computational calculations quantified these interactions at the molecular level, providing insights into the role of the zeolite framework and acid sites in a reaction involving alcohol or water OH and alkyl groups. This understanding contributes to the design of catalysts with tailored catalytic activity and selectivity.

## Results and Discussion

### C_1_–C_4_ Primary Alcohol Adsorption on Silicalite-1 (Si-MFI) and Siliceous Beta (Si-BEA)

Figure 1a,b shows the differential heat of adsorption of C_1_–C_4_ primary alcohols on Si-MFI and Si-BEA zeolites as a function of alcohol uptake at 323 K. The adsorption of each alcohol on Si-MFI and Si-BEA shows the differential heat of adsorption to be invariant with respect to the amount of sorbed alcohol. Figure 2a plots the differential heat of adsorption as a function of the alcohol carbon number and shows the heat of alcohol adsorption on Si-MFI and Si-BEA increases linearly by 11 and 10 kJ mol^–1^ per CH_*x*_ group, respectively (from 45 and 41 kJ mol^–1^ for methanol to 76 and 70 kJ mol^–1^ for 1-butanol). These values are in line with the previous studies on alkane adsorption, where each CH_*x*_ group contributes 10–12 kJ mol^–1^ and 8–10 kJ mol^–1^ to the heat of adsorption on **MFI** and **Beta** framework, respectively.^11^ The intercepts in Figure 2a allow quantifying the heat of adsorption contributed by interaction between the alcohol–OH group and the silica framework of the **MFI** and **Beta** channels, which are 35 kJ mol^–1^ (Si-MFI) and 30 kJ mol^–1^ (Si-BEA), respectively. The 5 kJ mol^–1^ higher value on Si-MFI compared to that on Si-BEA suggests a stronger interaction between the alcohol–OH and the **MFI** framework induced by the more constrained environment.

**Figure 1:**
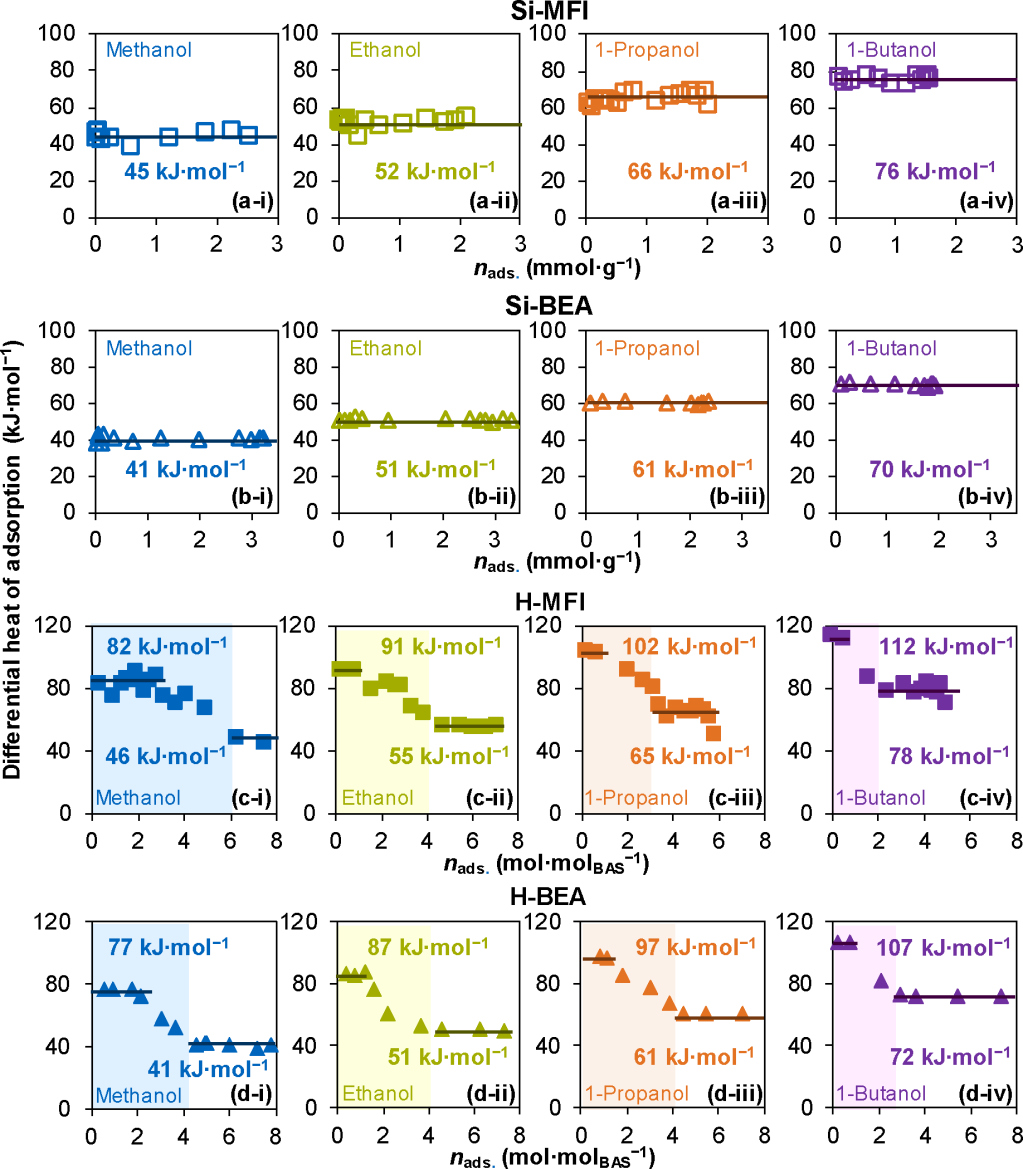
Heat of adsorption of C_1_–C_4_ primary alcohols on all samples. Differential heat of adsorption of C_1_–C_4_ primary alcohols as a function of alcohol uptake on (a-i)–(a-iv) Si-MFI and (b-i)–(b-iv) Si-BEA at 323 K. Differential heat of adsorption of C_1_–C_4_ primary alcohols as a function of adsorbed alcohol per BAS on (c-i)–(c-iv) H-MFI and (d-i)–(d-iv) H-BEA at 323 K.

**Figure 2:**
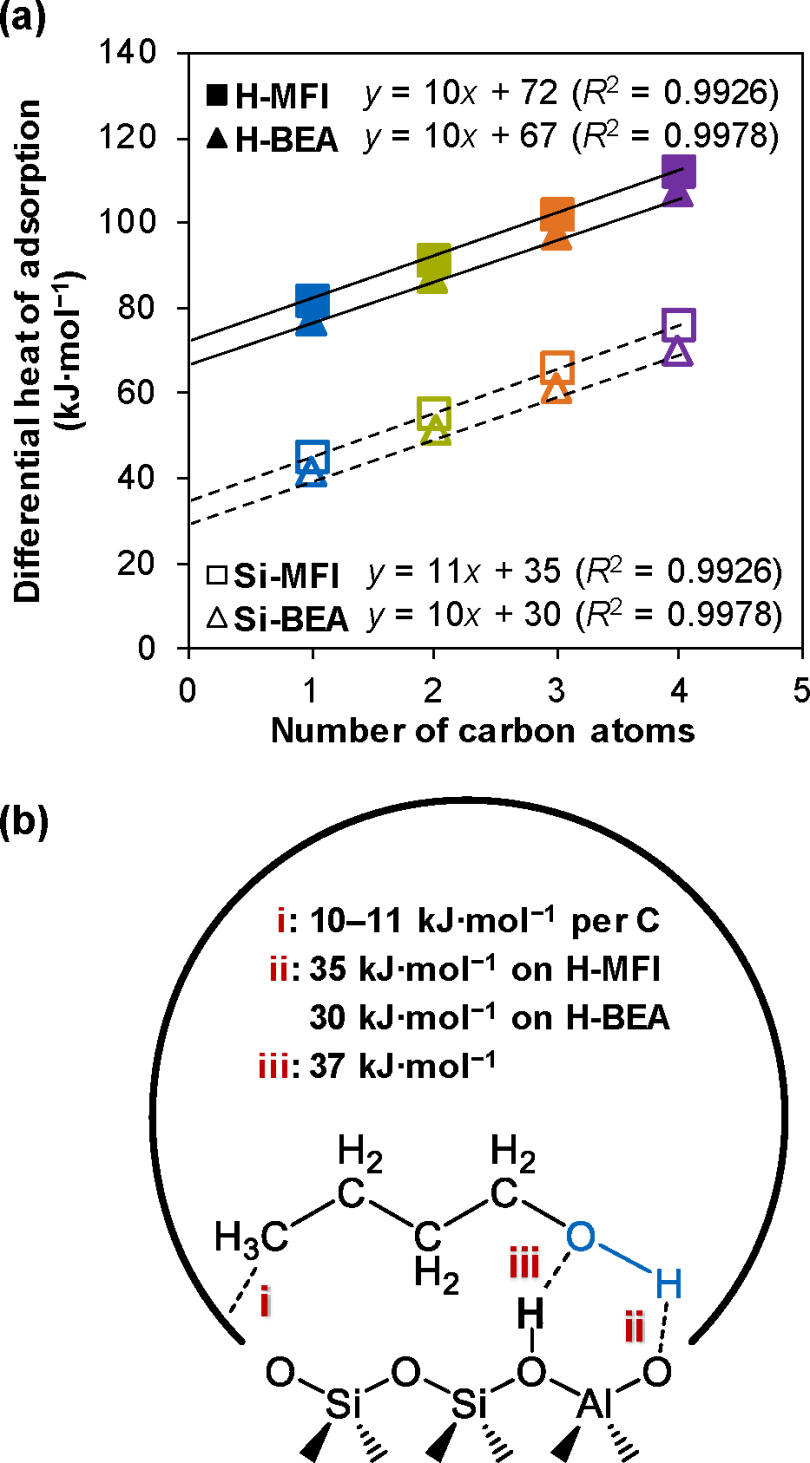
Differential heat of adsorption and energetic attribution of alcohol adsorption in zeolite. (a) Differential heat of adsorption of C_1_–C_4_ primary alcohols as a function of carbon atoms on Si-MFI (open square symbols), Si-BEA (open triangle symbols), H-MFI (solid square symbols), and H-BEA (solid triangle symbols) at 323 K. (The increment of the linear dependence on Si-MFI and Si-BEA indicates the interaction between each −CH_2_– (or −CH_3_) group and the zeolite framework, and the intercept value indicates the interaction between the alcohol–OH group and the zeolite framework; the increment of the linear dependence on H-MFI and H-BEA indicates the interaction between each −CH_2_– (or −CH_3_) group and the zeolite framework, and the intercept value indicates the interaction between the alcohol–OH group and the zeolite framework/BAS.) (b) Three kinds of interactions between a primary alcohol molecule (e.g., 1-butanol) and a BAS containing zeolite and their correlated contributions to the heat of adsorption.

To deepen our understanding of how zeolite channels interact with sorbed alcohol molecules, we investigated the sorption of C_1_–C_4_ primary alcohols in Si-MFI using IR spectroscopy and compared these results to the spectra in the gas phase and those adsorbed on silica (Figure 3). Si-MFI was selected for this analysis due to its highly crystalline and highly defect-free structure, as evidenced by the very low intensities of silanol group-related bands in the IR spectra (Figure S1). This allows us to isolate interactions between alcohol molecules and the pore walls [Si­(OSi)_4_], excluding contributions from the silanol groups. Corresponding results for Si-BEA are provided in Supplementary Note 1. In the following discussion, the term “pore wall” refers specifically to the closed Si sites as [Si­(OSi)_4_].

**Figure 3:**
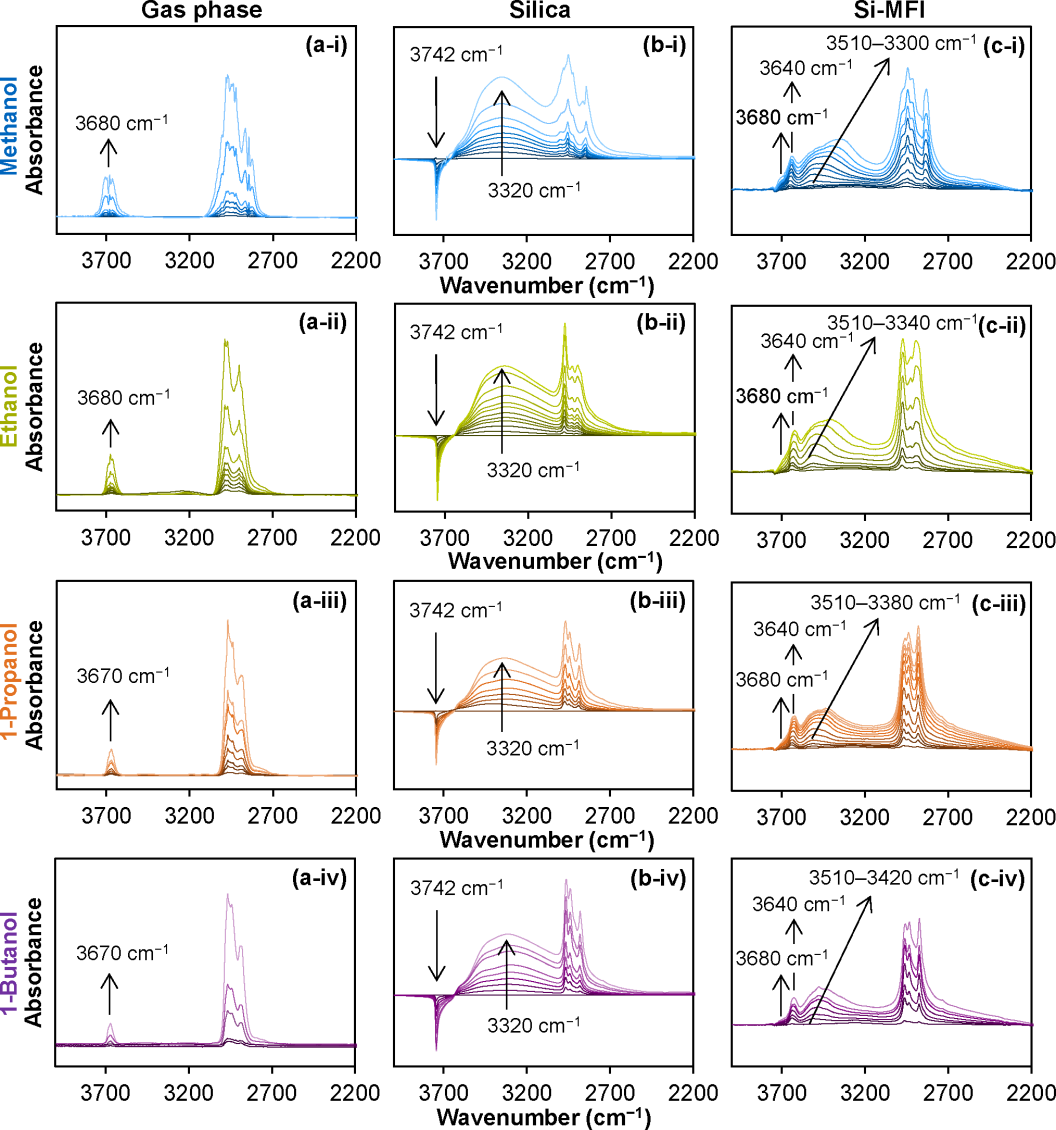
Infrared spectroscopy of C_1_–C_4_ primary alcohols. Infrared spectra of (a-i)–(a-iv) gas-phase C_1_–C_4_ primary alcohols, difference spectra of C_1_–C_4_ primary alcohols sorbed on (b-i)–(b-iv) silica (parent silica spectrum subtracted), and (c-i)–(c-iv) Si-MFI (parent Si-MFI spectrum subtracted). All spectra were recorded at 323 K under increasing pressure (1 × 10^–3^ to 20 mbar), with lighter colors representing higher pressures. Arrows indicate the trends in band changes.

In the gas-phase spectra (Figure 3a), the band at 3680 cm^–1^ is attributed to the OH stretching vibration of the unperturbed OH group of the alcohol. The bands between 2800 and 3000 cm^–1^ are attributed to the C–H vibrations of the alkyl groups. In contrast, sorbed alcohols on SiO_2_ (Figure 3b) do not exhibit a band for the free terminal OH. This absence is attributed to the interaction of the alcohol–OH group with the Si–OH groups, which not only leads to the negative band at 3742 cm^–1^ due to the perturbation of the Si–OH sites but also results in a band at 3320 cm^–1^, indicating hydrogen bonding within clusters of molecules (Figure 3b).^36,37^

Upon alcohol adsorption on Si-MFI, three bands appeared at 3680, 3640, and 3510 cm^–1^ in the O–H vibration range. The first band (3680 cm^–1^) is attributed to free alcohol–OH groups of nonadsorbed molecules in the gas phase, which only appeared at high alcohol pressure. The second (3640 cm^–1^) is assigned to alcohol molecules in the pores that do not interact with the pore walls. The shift of the free alcohol–OH group from 3680 cm^–1^ in the gas phase to 3640 cm^–1^ in Si-MFI is attributed to these weak interactions in the pore confinement.^38^ When interacting with the pore walls, the vibrational band of the free terminal alcohol–OH group shifts from 3640 to 3510 cm^–1^. This shift indicates a relatively strong interaction with the siliceous framework walls, likely enhanced by hydrogen bonding between alcohol molecules. As the alcohol concentration increased, the broad band gradually shifted to 3420–3300 cm^–1^. Paralleling the development of larger alcohol clusters, it points to a strengthening of the interactions with the silicate pore walls as a result of the polarization of the molecules in the hydrogen-bonded clusters.

### C_1_–C_4_ Primary Alcohol Adsorption on H-MFI and H-BEA

Figure 1c,d shows the differential heat of adsorption of C_1_–C_4_ primary alcohols on H-MFI and H-BEA zeolites as a function of adsorbed alcohol molecules per BAS. The monomolecular adsorption (*n*_ads._ ≤ 1 mol_alcohol_ mol_BAS_^–1^) results in a higher differential heat of adsorption, 82–112 kJ mol^–1^ and 77–107 kJ mol^–1^ for C_1_–C_4_ alcohols on H-MFI and H-BEA, respectively. The sorption enthalpy shows a linear relationship with the carbon number (Figure 2a), and the slope indicates that each CH_*x*_ group contributes 10 kJ mol^–1^, in agreement with their contribution with siliceous zeolites (Si-MFI and Si-BEA). On the other hand, the intercepts were 72 and 67 kJ mol^–1^ for H-MFI and H-BEA, respectively. Comparing the 35 and 30 kJ mol^–1^ values measured for siliceous zeolites, we attribute the 37 kJ mol^–1^ enthalpy increase (both H-MFI and H-BEA) specifically to the interaction between the alcohol–OH group and the BAS. Incidentally, although both H-MFI and H-BEA contain some silanol groups, alcohol molecules in the monomolecular adsorption regime interact exclusively with the BAS, not with the silanol groups (Figure S3). Therefore, interactions between sorbed alcohol and silanol groups can be excluded when comparing the monomolecular adsorption stage.

After the first molecule adsorbs, the heat of adsorption of C_1_–C_4_ primary alcohols on H-MFI and H-BEA continuously decreases with each additional sorbed molecule (*n*_ads._ > 1 mol_alcohol_ mol_BAS_^–1^) converging to a value similar to the alcohol adsorption enthalpy on siliceous zeolites. Prior to convergence, H-MFI and H-BEA are always higher than the corresponding heat of adsorption for the siliceous zeolite. This suggests the existence of additional intermolecular interactions between the adsorbed alcohol molecules in the presence of the BAS, indicating the formation of alcohol clusters around the BAS due to the strengthening of hydrogen bonding by the protonation of the alcohol. The size of the cluster of different alcohols is discussed in Supplementary Note 3.

In summary, Figure 2b illustrates three distinct interactions between the alcohol molecule and the zeolite, i.e., (i) the alkyl chain interacts with the zeolite pore (approximately 10 kJ mol^–1^ per carbon atom); (ii) the alcohol–OH group interacts with the zeolite pore (35 kJ mol^–1^ in the **MFI** framework and 30 kJ mol^–1^ in the **Beta** framework); and (iii) the alcohol–OH group interacts with the BAS (37 kJ mol^–1^ in both **MFI** and **Beta** frameworks).

### Relation between the Adsorption of the Alcohol–OH Group and Water

In our previous study, water adsorption on H-MFI showed that the first two sorbed water molecules interact strongly with the BAS, exhibiting an average heat of adsorption of approximately 71 kJ mol^–1^ (Figure 4a).^26–29,31^ This value aligns with the intercept of the linear relationship between the differential heat of adsorption of C_1_–C_4_ alcohols and the number of carbon atoms (Figure 4c), representing the interaction between the alcohol–OH group and the zeolite framework/BAS (interactions ii and iii in Figure 2b). In consequence, the OH groups of water are concluded to interact with a strength similar to the OH group of alcohols (C_0_OH). This is corroborated by the adsorption enthalpy of water on Si-MFI (33 kJ mol^–1^) (Figure 4b), identical to the intercept of the extrapolated correlation of the adsorption enthalpies and the carbon number of the alcohol (Figure 4c).

**Figure 4:**
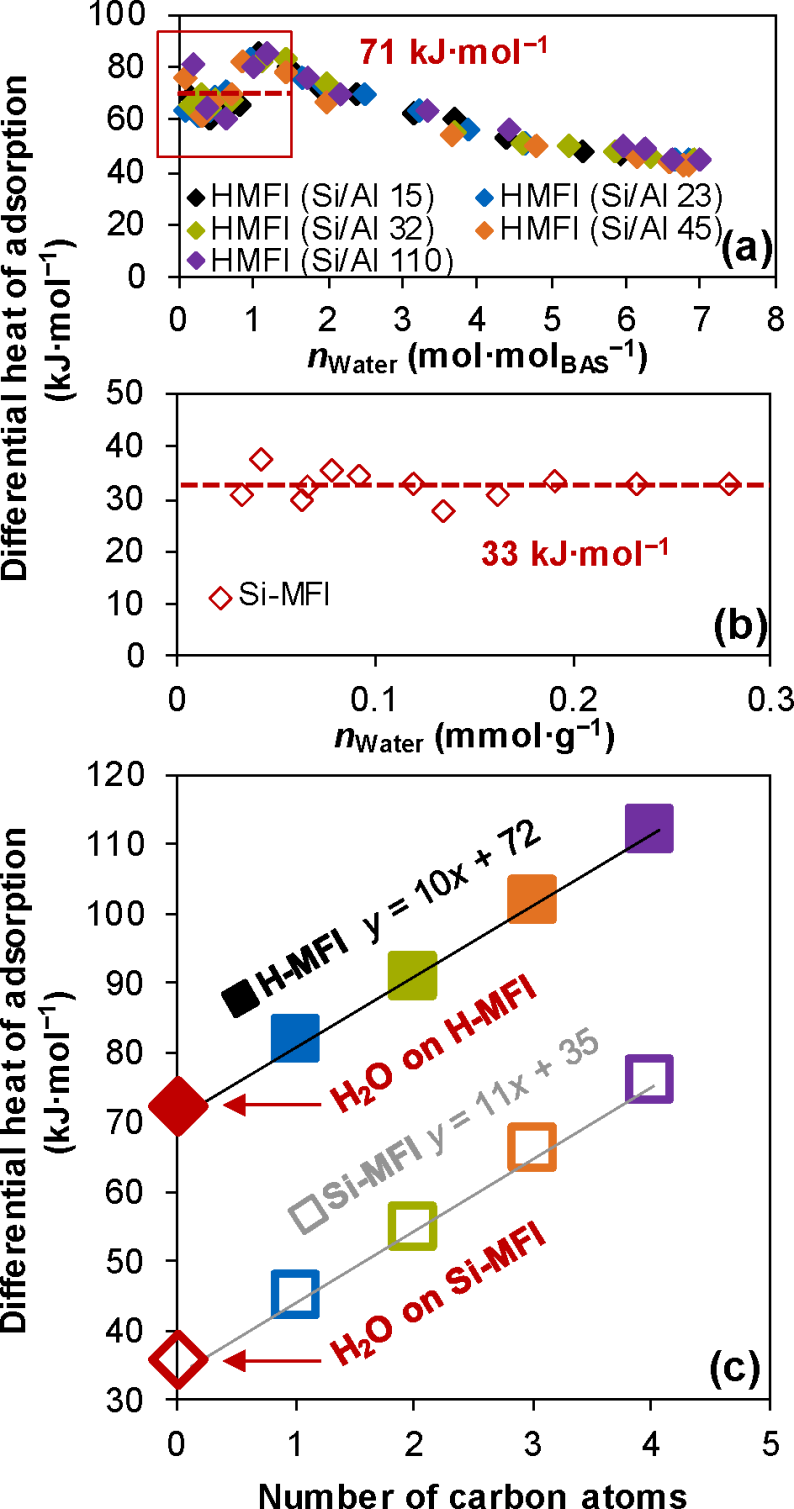
Differential heat of adsorption of water vs C_1_–C_4_ primary alcohols in MFI zeolites. Differential heat of adsorption of water as a function of adsorbed water on (a) H-MFI with different Si/Al ratios and (b) Si-MFI. (c) Differential heat of adsorption of water and C_1_–C_4_ primary alcohols as a function of carbon atoms on Si-MFI (open symbols) and H-MFI (solid symbols) at 298 K. (The heat of adsorption of water on H-MFI in (a) is adapted from ref 28. The heat of adsorption of water on H-MFI in (c) was calculated as the average value with *n*_Water_ ≤ 2 mol mol_BAS_^–1^).

It should be noted that Silicalite-1 (Si-MFI) is commonly regarded as ″hydrophobic″, i.e., only a small concentration of water can be adsorbed in the pores even in the presence of liquid water. The low concentration of water molecules in the pores stems from the fact that the enthalpy of condensation of water is higher (45 kJ mol^–1^) than the heat of adsorption of the OH group (33 kJ mol^–1^). This difference is due to the strong intermolecular hydrogen bonds in water (approximately 23.3 kJ mol^–1^),^39^ making it a unique sorbate compared to alcohols, whose heat of adsorption is generally higher than the condensation heat (Table S1). The more negative condensation enthalpy leads to very low concentrations of water in the pores of a perfect siliceous zeolite (OH group free) with the **MFI** structure. It is noticeable that the interaction between water and the “hydrophobic” pore is much stronger (33 kJ mol^–1^) than the one of alkyl groups (10–11 kJ mol^–1^). Thus, hydrogen bonding of the OH group of the alcohol (and of water) to the silica wall of the zeolite framework is stronger than the dispersive forces of the hydrocarbon groups. Clearly, the lower enthalpy gain with methanol and other alcohols upon condensation allows complete pore filling. It will be interesting to explore how mixtures of water with alcohols partition between the pores and the liquid, allowing in turn to establish the thermodynamic states of the solution constituents with and without the constraints of a porous sorbent and a catalyst.

### AIMD Simulations of Alcohol Adsorption on Si-MFI

To better understand the discrete interactions between alcohols and zeolites, we employed density functional theory (DFT) based on ab initio molecular dynamics (AIMD) simulations for the C_1_–C_4_ primary alcohols within the **MFI** frameworks. We generated trajectories for a single alcohol molecule within Si-MFI and H-MFI at 333 K. In the case of H-MFI, the simulation cell included only a single BAS.

Figure 5a shows the simulated adsorption enthalpy, −Δ*H*°_the._, of the alcohols in Si-MFI calculated by the quasi-harmonic approximation (QHA) method from the AIMD trajectories. −Δ*H*°_the._ increases linearly with increasing number of carbon atoms, the slope of which indicates that each CH_*x*_ contributes 9 kJ mol^–1^, and the *y*-intercept provides the alcohol–OH interaction with the zeolite framework as 37 kJ mol^–1^; the corresponding experimental values (Figure 2a) for Si-MFI are 11 and 35 kJ mol^–1^. DFT simulations underestimate the CH_*x*_ binding by 2 kJ mol^–1^ and the OH interaction by the same value. Considering the typical accuracy of DFT calculations,^40,41^ this is a very good agreement and strongly supports the validity of the structural analysis that follows. Figure 5b shows a linear correlation between experimental and theoretical enthalpy changes, with a slope of 0.86 and *R* ^2^ = 0.98, indicating that the theoretical results not only capture the overall trend but also closely match the adsorption enthalpy for the individual alcohols.

**Figure 5:**
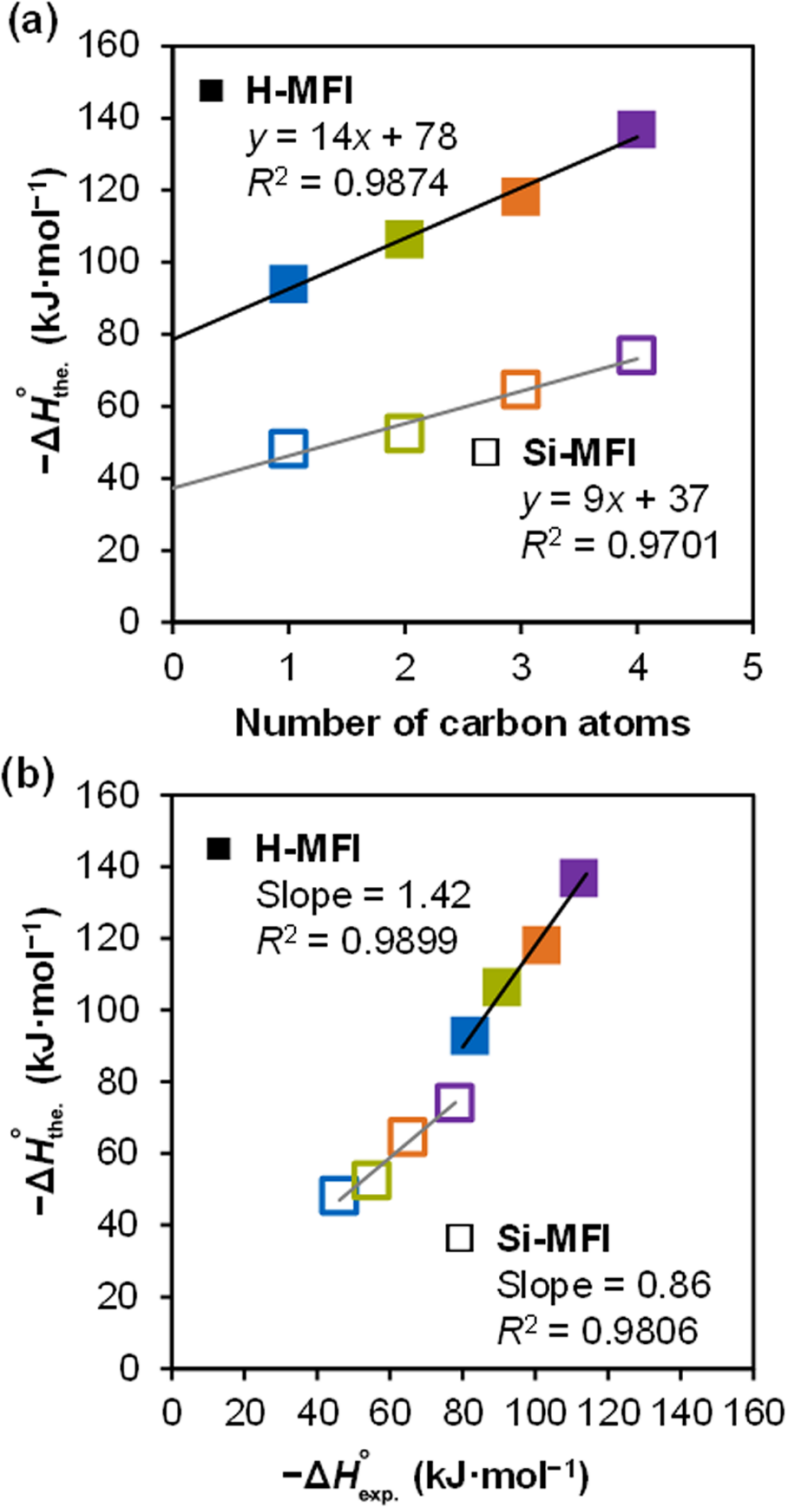
(a) Calculated standard enthalpy of adsorption, −Δ*H*°_ads_, for C_1_–C_4_ primary alcohols in Si-MFI and H-MFI, and (b) comparison between calculated enthalpy, – Δ*H*°_the._, and the experimentally measured differential heat of adsorption, −Δ*H*°_exp_.

We then computed the radial distribution function *g*(*R*) using the AIMD trajectories, which provide the average distances between pairs of atoms. In Figure 6a, *g*(*R*) is plotted for the distances between carbon atoms in the alcohol, C_A_, and the oxygen and silicon atoms of the zeolite framework, O_Z_ and Si. The *g*(*R*) values decrease with an increase in carbon numbers in the 3.0–5.5 Å distance range, while they increase in the 5.5–8.0 Å range (Figure 6a-ii). The free energy of the C_A_–Si/O_Z_ interaction was estimated by calculating the potential of the mean force (PMF) as *V*(*R*) = – *RT* ln­(*g*(*R*)) (Figure 6a-iii). From this, we see that despite the apparent changes in *g*(*R*) morphology the PMF experienced by each carbon atom is identical for C_1_–C_4_ (12.9 ± 0.37 kJ mol^–1^). This observation explains the linear increase in −Δ*H* with an increasing number of carbons, consistent with experimental results.

**Figure 6:**
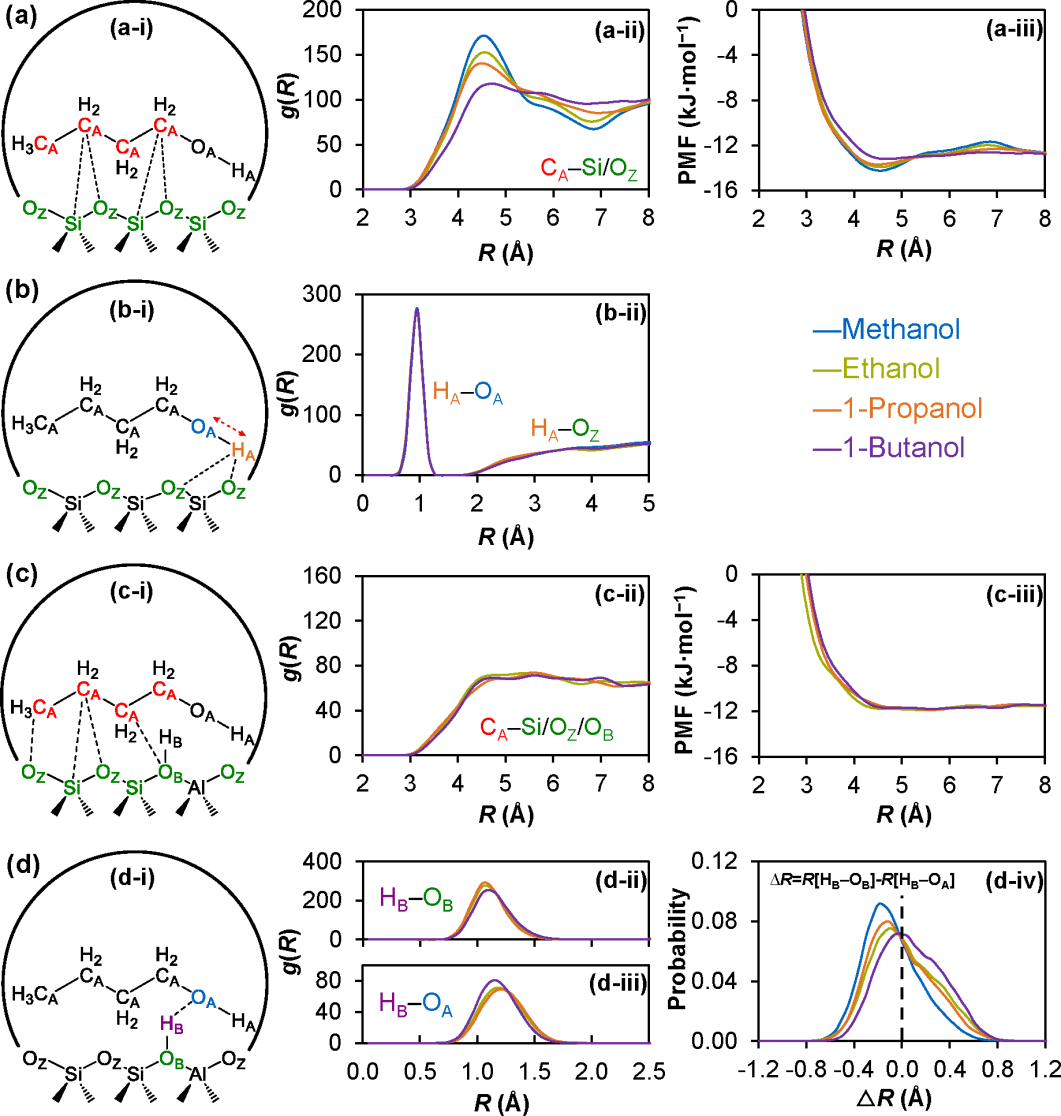
Interactions of C_1_–C_4_ primary alcohols in the MFI framework. In Si-MFI: (a-i) CH_*x*_ interactions of C_1_–C_4_ primary alcohols, (a-ii) radial distribution function, *g*(*R*), for C_A_, to Si and O_Z_, and (a-iii) potential of mean force (PMF) calculated as *V*(*R*) = – *RT* ln­(*g*(*R*)), which is an estimate of the free energy of the average interaction between C_A_ and Si/O_Z_. (b-i) OH interactions of C_1_–C_4_ primary alcohols in Si-MFI, (b-ii) *g*(*R*) for H_A_–O_A_, as well as the *g*(*R*) for H_A_–O_Z_. In H-MFI: (c-i) CH_*x*_ interactions of C_1_–C_4_ primary alcohols, (c-ii) *g*(*R*) for C_A_ and Si/O_Z_/O_B_, and (c-iii) PMF of the average interaction between C_A_ and Si/O_Z_/O_B_. (d-i) OH interactions of C_1_–C_4_ primary alcohols in H-MFI, (b-ii) *g*(*R*) for H_B_–O_B_, (b-iii) *g*(*R*) for H_B_–O_A_ for C_1_–C_4_, and (d-iv) probability distribution for the relative position of H_B_ between O_A_ and O_B_. The difference in the H_B_–O_B_ and O_A_–H_B_ distances, Δ*R*, is calculated as Δ*R* = *R*[H_B_ – O_B_] – *R*[H_B_ – O_A_] at each time step (0.5 fs), followed by a histogram of Δ*R* values. Positive values indicate that the BAS proton is primarily on O_A_ and negative on O_B_. (C_A_, O_A_, and H_A_ represent alcohol carbon, alcohol oxygen, and alcohol hydrogen, respectively; Si and O_Z_ represent framework silicon and oxygen, respectively; and H_B_ and O_B_ represent BAS proton and BAS oxygen, respectively).

Figure 6b depicts *g*(*R*), showing distances for the alcohol hydrogen–oxygen (H_A_–O_A_) and the alcohol hydrogen to the zeolite framework oxygen (H_A_–O_Z_) for C_1_–C_4_. The H_A_–O_A_ peak centered at about 1.0 Å is unchanged for C_1_–C_4_, showing that the carbon length has a negligible effect on the H_A_–O_A_ interaction. The H_A_–O_Z_ distances are similarly unaffected by the carbon length, with the shortest distance occurring at about 2.0 Å. Together, these two illustrate that the carbon chain length has no effect on both the intramolecular H_A_–O_A_ and intermolecular H_A_–O_Z_ interactions, explaining why the alcohol–OH group consistently contributes approximately 35 kJ mol^–1^ to the experimental adsorption enthalpy for C_1_–C_4_ alcohols.

### AIMD Simulations of Alcohol Adsorption on H-MFI

As with Si-MFI, we observe a linear relationship between the calculated −Δ*H* and carbon number for C_1_–C_4_ primary alcohols in H-MFI. A linear fit indicates that each CH_*x*_ contributes 14 kJ mol^–1^ and the alcohol–OH contributes 78 kJ mol^–1^ to the enthalpy of adsorption (Figure 5a). Overall, the −Δ*H*°_the._ trend strongly agrees with the experimental differential heat of adsorption; the AIMD description of the alcohol–OH contribution to Δ*H*°_ads._ differs by only 6 kJ mol^–1^ and the CH_*x*_ component by 4 kJ mol^–1^, relative to the experiment (Figure 2a). Comparing the experimental and theoretical adsorption enthalpies for C_1_–C_4_ (Figure 5b), we observe a strong linear relationship with a slope of 1.42 (*R* ^2^ = 0.9899), indicating a uniform overestimation of the calculated Δ*H*°_ads._. Initially, this suggests a discrepancy in the accuracy of the calculated Δ*H*°_ads._ in Si-MFI relative to H-MFI; however, it is crucial to emphasize that both exhibit remarkable agreement when considering the typical DFT error associated with describing adsorbate interactions using generalized gradient approximation (GGA) functionals.^40,41^ Previous studies on zeolite adsorption of alkanes with a similar methodology have found errors on the order of 3–5 kJ mol^–1^.^42^

For H-MFI, the presence of the BAS results in two distinct interactions of the alcohol carbons C_A_ with the zeolite oxygen, one for the zeolite framework, O_Z_, and another for the oxygen of the acid site, O_B_. Figure S6 plots the *g*(*R*) and illustrates that there is a distinction in the C_A_–O_B_ interaction for C_1_–C_4_, with each subsequent carbon added being further from O_B_. However, this effect is insignificant compared to the more abundant C_A_–O_Z_ interaction, which deviates little for C_1_–C_4_. This is clearly illustrated in Figure 6c-ii depicting the *g*(*R*) for C_A_ to all atoms of the zeolite framework (Si, O_Z_, and O_B_), which shows strong morphological agreement for C_1_–C_4_. Also, the PMF of Figure 6c-iii shows each C_A_ experiences an identical force from the zeolite framework (11.7 ± 0.16 kJ mol^–1^). Collectively, these observations elucidate why each CH_*x*_ equally contributes (experimental 9 kJ mol^–1^) to the heat of adsorption.

Depicted in Figure 6d is the interaction of the BAS proton (H_B_) with either the BAS oxygen (O_B_) or the alcohol oxygen (O_A_), along with the associated *g*(*R*). The alcohol O_A_ hydrogen bonds to H_B_ when interacting with the BAS. The *g*(*R*) of H_B_–O_B_ shows peak broadening from C_1_ to C_4_, with a tail stretching to longer distances as H_B_ is increasingly shared with the alcohol (Figure 6d-ii). In Figure 6d-iii, we see the H_B_-O_A_ distance shortening with the carbon number. The relative position of H_B_ between O_B_ and O_A_ is the most clearly represented using the Δ*R* probability distribution shown in Figure 6d-iv; here, negative values of Δ*R* indicate that H_B_ is closer to O_B_ than to O_A_ and vice versa for positive values. For C_1_, H_B_ is primarily on O_B_ with little probability of moving toward O_A_. For C_2_ and C_3_, H_B_ shifts toward O_A_ but remains primarily on the side of O_B_. For C_4_, H_B_ is nearly equally shared between the two oxygens, forming a Zundel-like proton structure. Figure S7 shows that these changes are predominantly due to a shortening of the length of the O_A_–H_B_ bond. This trend in Δ*R* follows the basicity of primary alcohols (see the p*K*_a_ in Table S1) and suggests a change in BAS interaction with increasing carbon number.

We expect the strength of the alcohol–OH interaction with the BAS to increase with increasing carbon number, which in turn reduces the acidity of the BAS–OH group.^43^ Using DFT, we observe variations in the interaction of C_1_–C_4_ primary alcohols with the BAS. The trend in adsorption enthalpies is better captured by a polynomial fit than a linear one (Figure S8). Given the uniformity of the alkyl interaction with the zeolite framework (Figure 6c-ii,iii), the observed nonlinearity is attributed to the influence of the alkyl chain on the OH group, i.e., the increasing base strength of the OH group of primary alcohols. It is worth noting, however, that the degree of nonlinearity is very small, with the polynomial fit differing from the linear fit by an average of only 1.67 kJ mol^–1^. Experimentally, this effect is not seen for primary alcohols. Yet, we do observe a decrease in the heat of adsorption of isomeric alcohols, which follows their basicity (see Supplementary Note 2). At present, the precise cause of this is unclear and is a subject for future investigation. We posit this to a pore confinement effect with changes in the strength of the O_A_/H_B_ interaction being canceled out by increased repulsive interactions with the wall, conformational changes in the alcohol, and other convoluting factors. Similar effects were observed for branched alkanes, which showed significantly weaker interactions with the zeolite lattice compared to *n*-alkanes.^25^ AIMD results indicate that variations in the BAS–OH binding strength are minimal and may be negligible, relative to these convoluting factors.

The presence of a BAS significantly complicates the computational modeling of interactions within the zeolite pores. While this study investigates a single BAS position, variations in BAS locations within the zeolite framework influence both acidity and adsorption behavior. Additionally, the orientation of adsorbed molecules plays a crucial role, as interactions along the “zigzag channels” or “straight channels” in H-MFI differ. These factors pose challenges for accurate modeling. Furthermore, nuclear quantum effects may influence the energetics of alcohol adsorption at the BAS, and prior studies have demonstrated their relevance at typical reaction temperatures.^44^ Despite these complexities, the present agreement between theory and experiment suggests that the ensemble of these effects as measured experimentally yields an outcome comparable to the model employed here.

## Conclusions

The present study provides a quantitative insight into the adsorption of C_1_–C_4_ alcohols and water on **MFI** and **Beta** frameworks by gravimetry, calorimetry, and theory. Correlations between the differential heat of adsorption of primary alcohols and the carbon number on siliceous and BAS containing frameworks reveal three distinct interactions between the alcohol molecules and the zeolite: (i) the interaction between the alkyl chain and the zeolite pore (approximately 10 kJ mol^–1^ per carbon atom), (ii) the interaction between the 1-alcohol–OH group and the zeolite pore (35 kJ mol^–1^ in the **MFI** framework and 30 kJ mol^–1^ in the **Beta** framework), and (iii) the interaction between the 1-alcohol–OH group and the BAS (37 kJ mol^–1^ in both **MFI** and **Beta** frameworks). Dipole–dipole interactions between the alkyl chain and the BAS, at only 10 kJ mol^–1^, contribute to approximately one-third of the adsorption energy and can be neglected at the near-ambient temperatures in this study. The adsorption of water is nearly identical to the sorption enthalpies attributed to the OH group of the alcohols. The results demonstrate that hydrogen bonding to the surface is significantly stronger than the interaction between CH_*x*_ groups and the pore walls. The remarkable agreement between theory and experiment confirms that the interactions between each CH_*x*_ moiety and the zeolite framework remain invariant, with respect to the carbon number for C_1_–C_4_ alcohols. Similarly, the interaction between the alcohol hydroxyl group and the MFI zeolite framework also remains unchanged.

These findings provide fundamental insights into intermolecular forces, surface interactions, and confinement effects of zeolite-alcohol systems. Understanding how polar and nonpolar groups interact with zeolite pores will provide insights into catalytic reactions such as dehydration, dehydrogenation, and alkylation. This knowledge can be utilized to design more efficient catalytic processes in the petrochemical, pharmaceutical, and fine chemical industries.

## Materials and Methods

### Materials

Si-MFI (Silicalite-1) and H-MFI (H-ZSM-5 with Si/Al = 45) with the **MFI** framework were obtained from *Clariant*. Si-BEA (siliceous) and H-BEA (Si/Al = 50) with the **Beta** framework were synthesized in the presence of fluoride following the procedure in a previous publication.^45^ In brief, **Beta** zeolite was prepared by hydrothermal synthesis at 140 °C, with a gel composition of SiO_2_:*x*Al_2_O_3_:(0.54 + 2*x*) TEAOH:(0.54 + 2*x*) HF:(7 + 2*x*) H_2_O. Subsequently, the collected material was washed and filtrated with deionized water until the pH was neutral. To decompose the residues, e.g., organic cation (TEA^+^), and remove fluoride anions, the dried material was calcined at 773 K for 2 h in synthetic air. Methanol (99.8%), ethanol (99.8%), 1-propanol (99.9%), 2-propanol (99.5%), 1-butanol (99.8%), 2-butanol (99.5%), and *iso*-butanol (≥99%) were purchased from *Sigma-Aldrich* and used as received.

### Zeolite Characterization

The surface area and pore volume of the zeolite were determined by nitrogen physisorption. The N_2_ adsorption isotherms were measured at 77 K using a *Porous Materials Inc.*. automatic *Sorptometer*. The zeolites were degassed in vacuum (<10^–3^ mbar) at 623 K overnight before each measurement. Surface area was calculated by applying the Brunauer–Emmett–Teller theory, and the t-plot method was used to determine the micropore volumes.

Acid site concentration on H-MFI and H-BEA was determined by the IR spectroscopy of adsorbed pyridine on a *PerkinElmer 2000* spectrometer with a resolution of 4 cm^–1^. The sample was shaped into a self-supporting wafer and activated under vacuum (10^–6^ mbar) at 723 K for 1 h. After cooling to 423 K, the sample was exposed and equilibrated with 0.1 mbar of pyridine vapor for 30 min with subsequent outgassing for 1 h. A spectrum of the chemisorbed pyridine was recorded thereafter. For quantification of acid site concentration, molar extinction coefficients of 0.73 and 0.96 cm μmol^–1^ were used for the band of pyridine on the Bro̷nsted acid site (at around 1545 cm^–1^) and on the Lewis acid sites (at around 1450 cm^–1^), respectively.

### Adsorption Measurements

The adsorption of alcohols and water on all zeolite samples was measured gravimetrically and calorimetrically on a microbalance in a *Seteram TG-DSC 111* calorimeter connected to a high-vacuum system. After pretreatment of an approximately 20 mg sample at 723 K for 1 h under vacuum (*p* < 10^–4^ mbar) with a heating rate of 10 K min^–1^, the sample was cooled to 323 K. Afterward, the adsorbate was introduced into the system by controlled dosing. The adsorbed alcohol was determined in small pressure steps from 1 × 10^–3^ to 6–120 mbar (varying due to the differing vapor pressures of each alcohol) at 323 K. The adsorbed water was determined in small pressure steps from 1 × 10^–3^ to 20 mbar at 298 K. The adsorbate uptake was determined by the increase of sample weight, and the released heat was obtained by integration of the heat flux signal.

IR spectroscopy of alcohols in the gas phase and alcohols adsorbed on silica and Si-MFI was used to identify the interaction of the alcohol molecules and the interacting surface. All spectra were collected at 323 K on a Vertex 70 spectrometer from Bruker Optics at a resolution of 4 cm^–1^. Sample wafers were loaded into a homemade IR cell connected to a vacuum system. It was first pretreated in vacuum (*p* < 10^–5^ mbar) at 723 K for 1 h and cooled to 323 K. Alcohols were then introduced into the system by controlled dosing. The adsorbed alcohols were determined in small pressure steps from 1 × 10^–2^ to 10 mbar.

### Computational Methods

We performed DFT-based AIMD simulations by modeling C_1_–C_4_ alcohol in **MFI** zeolite (same nomenclature as in the experiment: Si-MFI represents the zeolite without the BAS proton analogizing Silicalite-1, H-MFI represents the zeolite with the BAS proton analogizing H-ZSM-5). DFT calculations were performed under periodic boundary conditions with the GGA using the CP2K computational package.^46,47^ The Perdew–Burke–Ernzerhoff exchange correlation functional^48^ was used in conjunction with the dispersion corrections from the DFT-D3 method of Grimme et al.^49^ Valence electrons were described using double-ζ quality basis sets^50^ and core electrons with norm-conserving pseudopotentials.^51^ Electrostatic terms were augmented with an auxiliary plane-wave basis set with a 400 Ry cutoff. The optimized cell was 20.022 × 19.899 × 13.383 Å^3^ for both Si-MFI and H-MFI. Based on the large size of this cell, Γ-point approximation was employed for Brillouin zone integration.

AIMD simulations were performed within the canonical NVT ensemble at 333 K with a time step of 0.5 fs and a Nosé–Hoover thermostat chain with a frequency of 4000 cm^–1^. Each simulation trajectory contained a single alcohol molecule (C_1_–C_4_) within the zeolite framework. For H-MFI only, one BAS is present. To ascertain reliable statistical properties, we collected well-equilibrated data of 120–200 ps by discarding the initial 30–60 ps of data. The lowest energy structure of each system was determined through slow temperature annealing simulations followed by geometry optimization with TZV2PX basis sets. Structural characteristics were analyzed via plots of radial distribution functions, *g*(*R*), which show distances between atomic pairs. The adsorption enthalpy was calculated by employing a QHA method, for which the vibrational density of states was calculated using the Fourier transform of the velocity autocorrelation function.^52–54^

## Supplementary Material



## Notes

Supplementary Note 1, additional characterization of C_1_–C_4_ primary alcohol adsorption; Supplementary Note 2, isomeric alcohol adsorption—influence of basicity; Supplementary Note 3, cluster formation of adsorbed alcohol molecules on BAS; Supplementary Note 4, additional figures and tables; and references (PDF)

## #

Chemical Science Division, Oak Ridge National Laboratory, Oak Ridge, Tennessee 37830, United States
