# Supplementary material for: Interactions of Polar and Nonpolar Groups of Alcohols in Zeolite Pores
Source: J Am Chem Soc. 2025 Jul 12;147(29):26049–59. doi: 10.1021/jacs.5c09340 (PMC12291437; doi:10.1021/jacs.5c09340)
Supplement: Supplementary file 1 [file ja5c09340_si_001.pdf]

Interactions of polar and nonpolar groups of alcohols in zeolite pores

Ruixue Zhao[1,*], Sungmin Kim[2,*], Mal-Soon Lee[2,*], Benjamin A. Jackson[2], Fuli Deng[1], Xiaomai Chen[1], Cong Zhou[2], Konstantin Khivantsev[2], Yue Liu[1,3], Vassiliki-Alexandra Glezakou[2,#], Roger Rousseau[2,#], Johannes A. Lercher[1,2,*]

[1] *Department of Chemistry and Catalysis Research Center, Technical University of Munich, Lichtenbergstrasse 4, 85748 Garching, Germany.*

[2] *Institute for Integrated Catalysis, Pacific Northwest National Laboratory, P.O. Box 999, Richland, WA99352, USA.*

[3] *Shanghai Key Laboratory of Green Chemistry and Chemical Processes, School of Chemistry and Molecular Engineering, East China Normal University, Shanghai, 200062, PR China.*

[#] *Current address: Chemical Science Division, Oak Ridge National Laboratory, Oak Ridge, TN 37830, USA.*

Content

Supplementary Note 1. Additional characterization of C_1–C_4 primary alcohols adsorption **2**

Figure S1. Infrared spectroscopy of all the samples. 2

Figure S2. Infrared spectroscopy of C_1–C_4 primary alcohols on Si-BEA. 2

Figure S3. Infrared spectroscopy of methanol and 1-butanol sorbed on H-MFI and H-BEA. 3

Supplementary Note 2. Isomeric alcohols adsorption — influence of basicity **4**

Figure S4. Heat of adsorption of isomeric C_1–C_4 alcohols on all samples. 4

Table S1. Differential heat of adsorption of C_1–C_4 alcohols on H-MFI, Si-MFI, H-BEA and Si-BEA at 323 K and the condensation heat ($-\Delta H^{o}_{vap.}$), gas basicity, proton affinity and pK_a of C_1–C_4 alcohols. 5

Figure S5. Isomeric alcohols adsorption on all samples. 5

Supplementary Note 3. Cluster formation of adsorbed alcohol molecules on BAS **5**

Table S2. Estimation of the number of alcohol molecules involved in the cluster formed at the BAS and the correlated volume and radius of the cluster determined from Figure 3 and Figure S4. 6

Supplementary Note 4. Additional figures and tables **6**

Table S3. Physicochemical properties of zeolite samples. 6

Figure S6. Radial distribution function of C_1–C_4 primary alcohols in H-MFI. 6

Figure S7. Bond lengths of O_B–H_B (solid lines) and O_A–H_B (dashed lines) for C_1–C_4 primary alcohols in H-MFI. 7

Figure S8. Polynomial fits of the differential heat of adsorption of C_1–C_4 primary alcohols as a function of the number of carbon atoms. 7

REFERENCES **7**

Supplementary Note 1. Additional characterization of C_1–C_4 primary alcohols adsorption

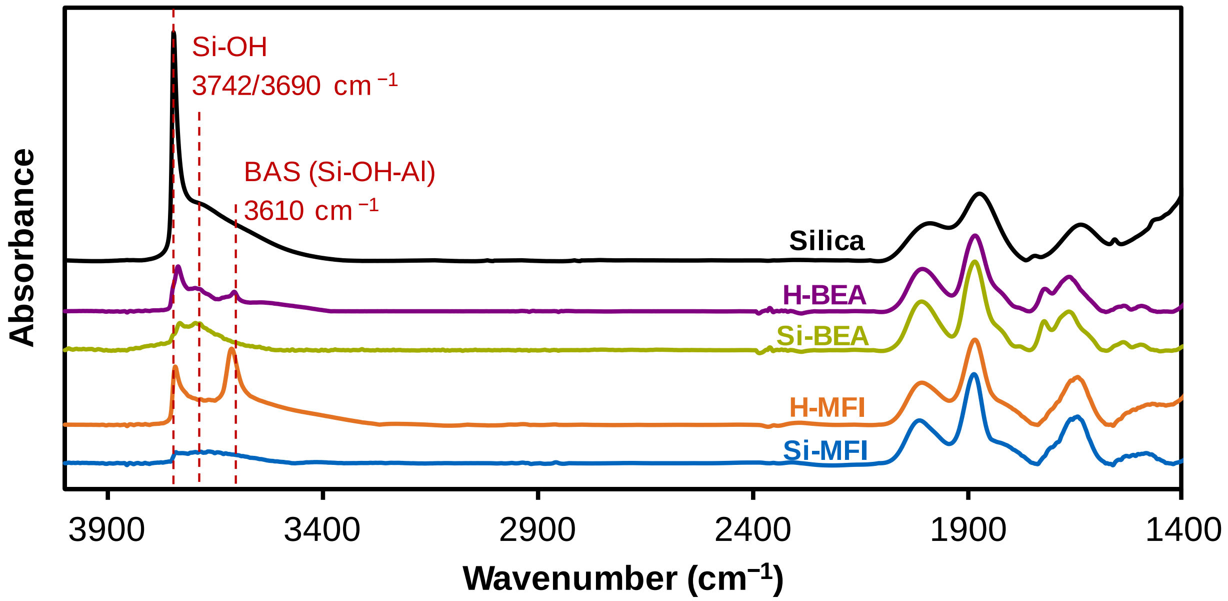

Figure S1. Infrared spectroscopy of all the samples. Infrared spectroscopy of Silica (black), Si-MFI (blue), H-MFI (orange), Si-BEA (green), and H-BEA (purple). Samples were activated in vacuum at 723 K for 1 h. All spectra were recorded at 323 K. For comparison, all spectra were normalized to the Si–O overtone peaks between 1700–2000 cm^{-1}.

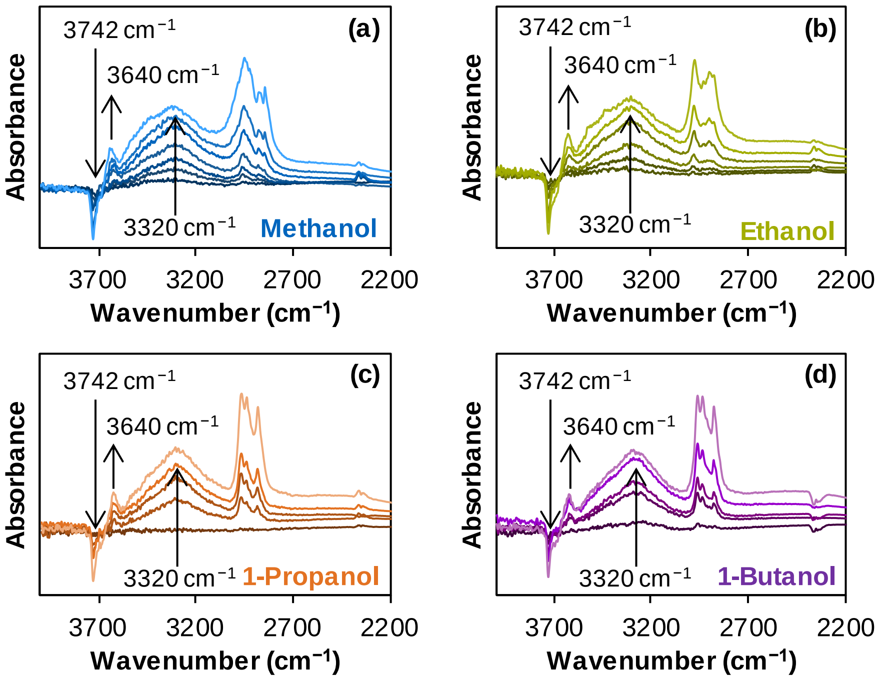

Figure S2. Infrared spectroscopy of C_1–C_4 primary alcohols on Si-BEA. Difference spectra of (a) methanol, (b) ethanol, (c) 1-propanol, and (d) 1-butanol sorbed on Si-BEA (parent Si-BEA spectrum subtracted). All spectra were recorded at 323 K under increasing pressure (1×10^{-3} to 20 mbar), with lighter colors representing higher pressures. Arrows indicate the trends in band changes.

The IR spectra presented in **Figure S1** reveal differences in surface hydroxyl features among the investigated samples. Silica typically exhibits broad bands in the 3400–3750 cm^{-1} region, corresponding to surface silanol (Si–OH) groups. In contrast, Si-MFI shows minimal silanol-related bands due to its highly crystalline and defect-free structure. However, Si-

BEA displays noticeable silanol bands, which are likely associated with structural defects or external surface terminations. Both H-MFI and H-BEA exhibit bands around 3610 cm^{-1}, characteristic of Brønsted acid sites (BAS, Si–OH–Al), in addition to remaining silanol signals.

Upon alcohol adsorption on Si-BEA (**Figure S2**), a band appeared at 3640 cm^{-1}, similar to the case observed for Si-MFI (**Figure 3c**), which corresponds to alcohol molecules in the pores but not interacting with the pore walls. However, as previously discussed in **Figure S1**, Si-BEA contains a greater number of silanol groups than the defect-free Si-MFI. As a result, the interaction between the sorbed alcohol with the zeolite framework are primarily governed by the interaction with Si–OH groups. This leads to a negative band at 3742 cm^{-1}, indicating perturbation of the Si–OH groups, and the appearance of a band at 3320 cm^{-1}, similar to the adsorption on SiO_2 (**Figure 3b**). Nevertheless, this interaction is not significantly stronger than that with a closed Si site in the framework as [$Si(OSi)_4$] according to the identical heat upon adsorption (**Figure 1b**).

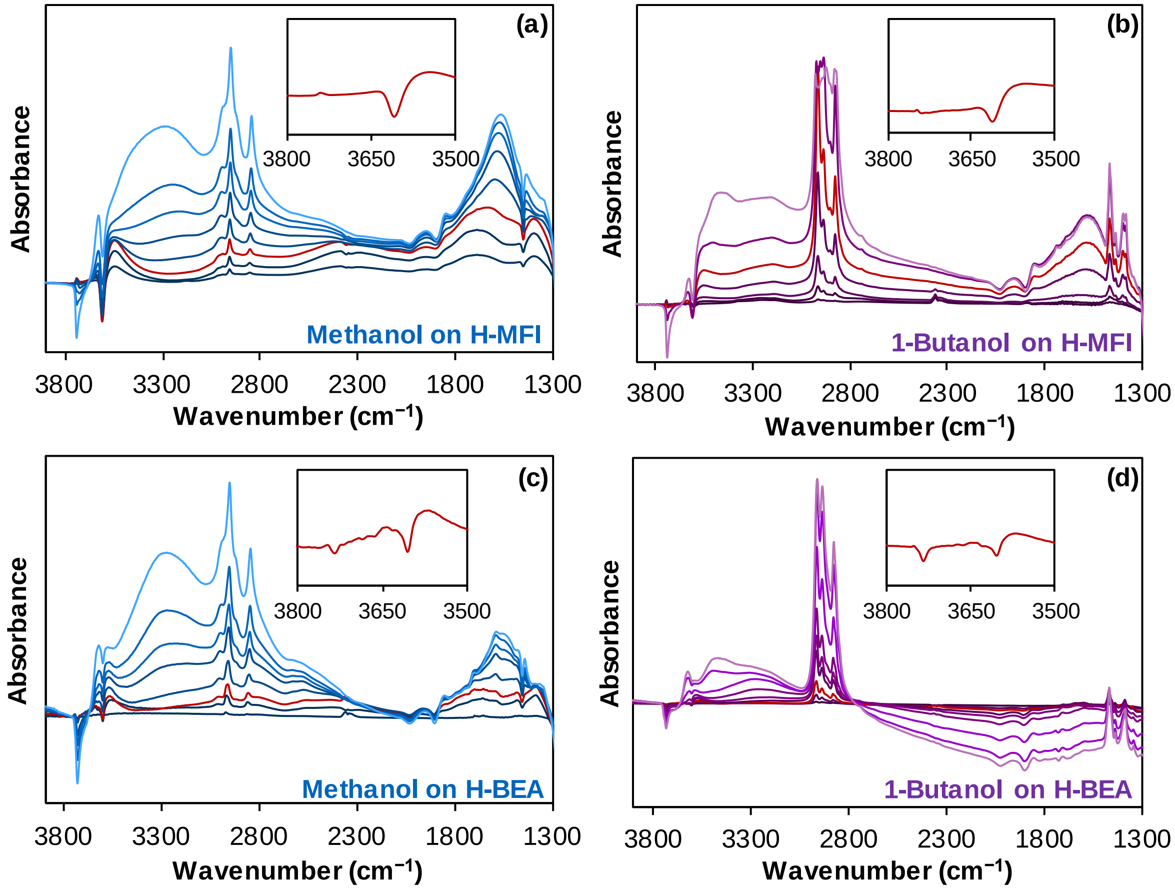

Figure S3. Infrared spectroscopy of methanol and 1-butanol sorbed on H-MFI and H-BEA. Difference spectra of methanol sorbed on (a) H-MFI, (c) H-BEA, and 1-butanol sorbed on (b) H-MFI, (d) H-BEA (parent H-MFI/H-BEA spectrum subtracted). Spectra shown in red correspond to alcohol uptake of $n_{ads.} \approx 1$ $mol_{alcohol} \cdot mol_{BAS}^{-1}$ at the monomolecular adsorption region. All spectra were recorded at 323 K under increasing pressure (1×10^{-3} to 20 mbar), with lighter colors representing higher pressures.

Methanol and 1-butanol were adsorbed on H-MFI and H-BEA, as shown in **Figure S3**. On both samples, alcohols initially interacted with the BAS, as evidenced by the gradual decrease in the band intensity at 3610 cm^{-1}, corresponding to BAS, until the loading reaches approximately 1 mol alcohol per mol BAS ($n_{ads.} \approx 1$ $mol_{alcohol} \cdot mol_{BAS}^{-1}$). Thereafter, the band at around 3740 cm^{-1} attributed to silanol (Si–OH) groups began to decrease, indicating that additional alcohol molecules interacted with the silanol groups. This observation is consistent with our previous findings on water adsorption in H-MFI, where water molecules do not interact with Si–OH groups at low water loadings.[1]

Supplementary Note 2. Isomeric alcohols adsorption — influence of basicity

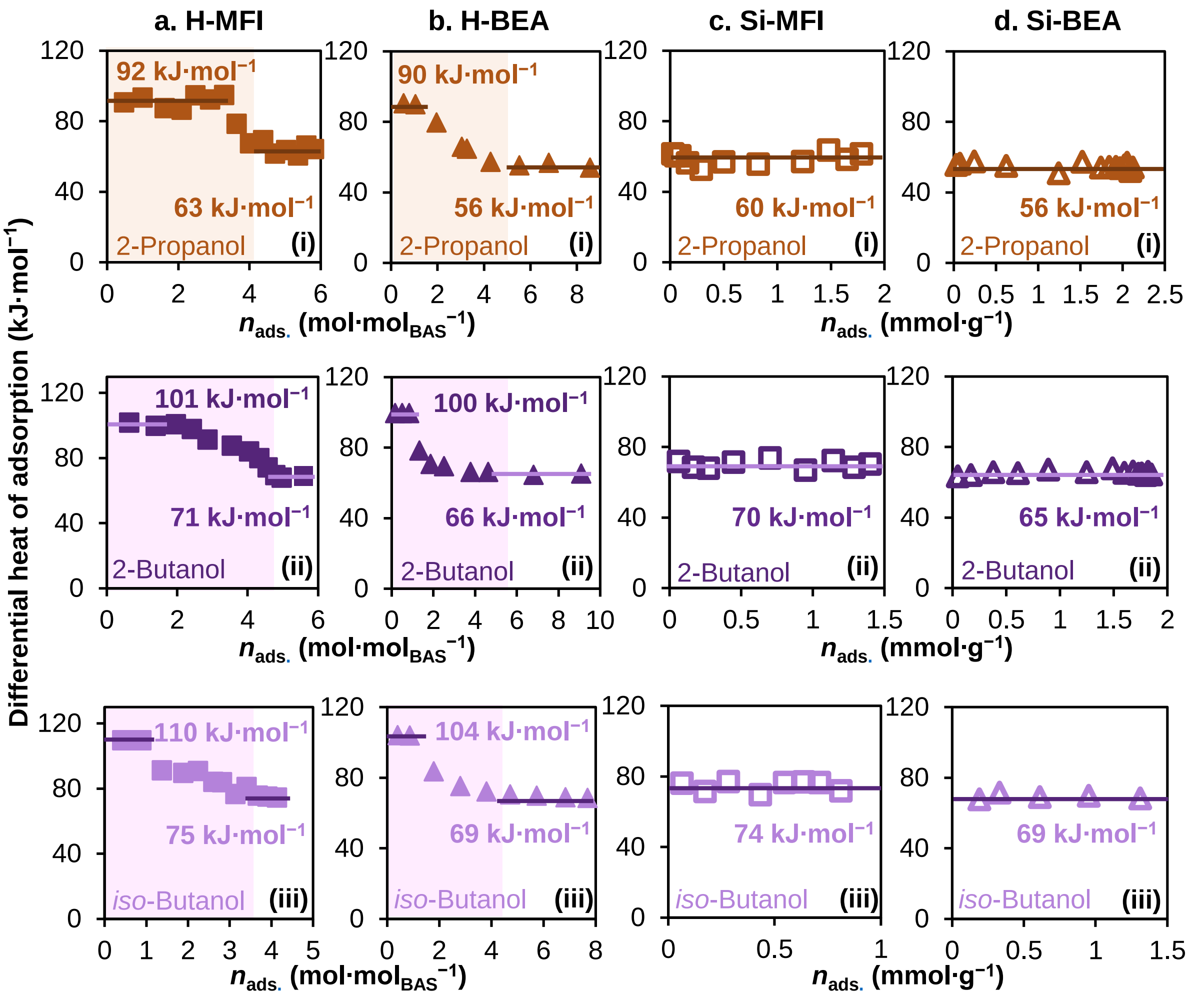

Figure S4. Heat of adsorption of isomeric C_1–C_4 alcohols on all samples. Differential heat of adsorption of 2-proponal (dark orange), 2-butanol (dark purple) and *iso*-butanol (light purple) as a function of adsorbed alcohol per BAS on (a-i)–(a-iii) H-MFI (solid square), (b-i)–(b-iii) H-BEA (solid triangle), (c-i)–(c-iii) Si-MFI (open square) and (d-i)–(d-iii) Si-BEA (open triangle) at 323 K.

We address how the basicity and isomeric structure of alcohol affects the heat of adsorption on H-MFI and H-BEA with isomeric alcohols such as 2-propanol, 2-butanol and *iso*-butanol (**Figure S4**). The heat of monomolecular alcohol adsorption on H-MFI and H-BEA is tabulated in **Table S1**. Isomeric propanol (2-propanol) and butanol (*iso*-butanol and 2-butanol) show lower differential heat of adsorption compared to the corresponding primary alcohols. In particular, the differential heat of adsorption of propanol shows the trend of 1-propanol > 2-propanol and butanol shows the trend of 1-butanol > *iso*-butanol > 2-butanol. The changes of the differential heat of adsorption of isomeric alcohols are in line with the increase in the basicity of the gas phase alcohols as following: 1-propanol > 2-propanol and 1-butanol > *iso*-butanol > 2-butanol (**Table S1**). It may be argued that the position of the alcohol–OH group at a secondary or tertiary carbon weakens the interaction between the alcohol molecule and the zeolite due to steric constraints. At present, the precise cause of this is unclear and is subject for future investigation. **Figure S5-a** plots the differential heat of adsorption of the secondary alcohols (2-propanol and 2-butanol) in the zeolites as a function of the number of carbon atoms. The heat of adsorption for the alkyl chains is comparable to the primary and secondary alcohols (approximately 10 kJ·mol^{-1} per carbon atom). However, the intercept values, which are lower for secondary alcohols as show in **Figure S5-b**, suggest that interactions of the secondary alcohol OH with the zeolite pores (type ii in **Figure 2b** and **Figure S5-b**) or the BAS (type iii in **Figure 2b** and **Figure S5-b**), are weaker compared to the primary alcohols. Especially, the interaction with the zeolite pores (type ii in **Figure 2b** and **Figure S5-b**) is weakened by 4–5 kJ·mol^{-1} for a secondary alcohol in both **MFI** and **Beta** frameworks, with **MFI** showing 5

kJ·mol^{-1} higher than **Beta** due to its more confined pore structure. Furthermore, the interaction with the BAS (type iii in **Figure 2b** and **Figure S5-b**) is weakened by 2–3 kJ·mol^{-1} for secondary alcohols (approximately 35 kJ·mol^{-1} for both **MFI** and **Beta** framework), maintaining similar values for both H-MFI and H-BEA. This is in accordance with the primary alcohols, which show equivalent enthalpy (approximately 37 kJ·mol^{-1} for both **MFI** and **Beta** framework), indicating that this interaction (type iii) is barely influenced by the framework.

Table S1. Differential heat of adsorption of C_1–C_4 alcohols on H-MFI, Si-MFI, H-BEA and Si-BEA at 323 K and the condensation heat ($-\Delta H^{o}_{vap.}$), gas basicity, proton affinity and pK_a of C_1–C_4 alcohols.

	Differential heat of adsorption (kJ·mol^{-1})				$-\Delta H^{o}_{vap.}$ [b]	Gas basicity[2]	Proton affinity[2]	pK_a (25 ℃)[c]	
	H-MFI[a]	Si-MFI	H-BEA	Si-BEA	(kJ·mol^{-1})	(kJ·mol^{-1})	(kJ·mol^{-1})	In water	In DMSO[d]
Water	71 ± 8	33 ± 3	-	-	42.9 ± 1.4 [3]	660.0	691	14.00 [4]	32 [5]
Methanol	82 ± 4	45 ± 3	77 ± 0	41 ± 1	37.6 ± 0.5	724.5	754.3	15.07 [6-8]	29.0 [5]
Ethanol	91 ± 1	52 ± 3	87 ± 1	51 ± 1	42.3 ± 0.4	746	776.4	15.83 [6-8]	29.8
1-Propanol	102 ± 1	66 ± 3	97 ± 1	61 ± 1	47 ± 1	756.1	786.5	15.92 [6-8]	
2-Propanol	91 ± 1	60 ± 3	90 ± 0	56 ± 2	45 ± 3	762.6	793.0	16.09 [6, 8]	30.25 [5]
1-Butanol	112 ± 1	76 ± 2	107 ± 0	70 ± 1	52 ± 3	758.9	789.2	15.87 [6-8]	
iso-Butanol	110 ± 0	74 ± 3	104 ± 0	69 ± 1	51 ± 1	762.2	793.7	15.91 [7]	
2-Butanol	101 ± 1	70 ± 2	100 ± 0	65 ± 1	48 ± 5	784.6	815	17.69 [7, 8]	

a The differential heat of monomolecular adsorption.
b Enthalpy of vaporization obtained from ref[9] excluding water.
c The average number calculated from the related references.
d DMSO: dimethyl sulfoxide

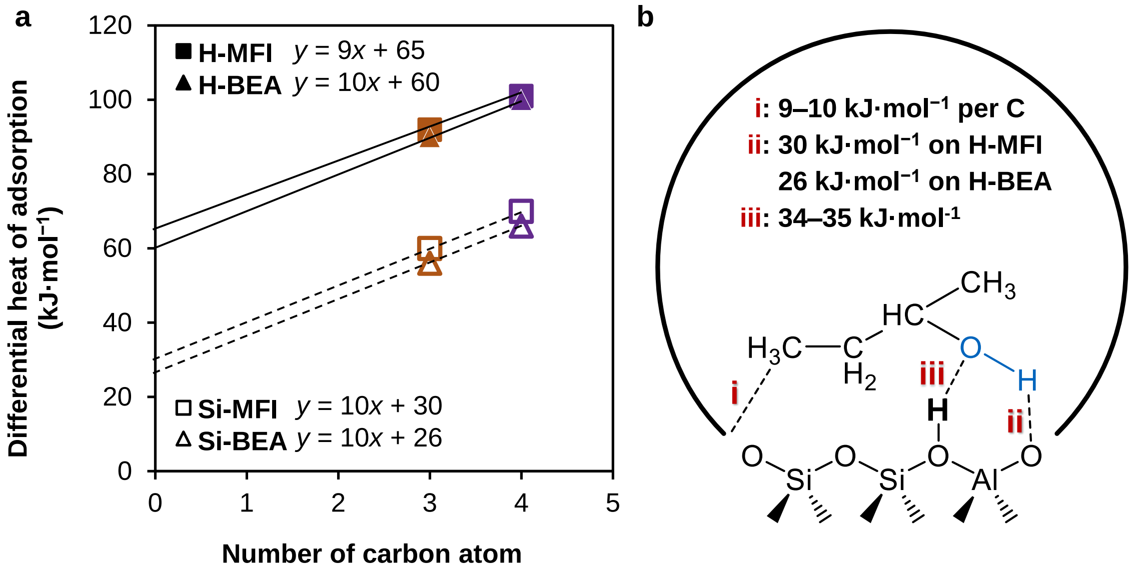

Figure S5. Isomeric alcohols adsorption on all samples. (a) Differential heat of adsorption of 2-propanol (dark orange) and 2-butanol (purple) as a function of carbon atoms on H-MFI (solid squares), H-BEA (solid triangles), Si-MFI (open squares) and Si-BEA (open triangles) at 323 K; (b) Three kinds of interaction between a secondary alcohol molecule (*i.e.* 2-butanol) and a BAS-containing zeolite and their correlated contributions to the heat of adsorption.

Supplementary Note 3. Cluster formation of adsorbed alcohol molecules on BAS

As discussed above and shown in **Figures 1** and **Figures S4**, we found a gradual decrease of the differential heat of adsorption after the monomolecular adsorption of alcohols on BAS (n_{ads} = 1 mol$_{alcohol}$·mol$_{BAS}^{-1}$). This suggests the formation of alcohol clusters around the BAS due to the additional inter-molecular interaction in the presence of BAS. Hence, we can estimate the cluster size, this is represented by the colored backgrounds in **Figures 1** and **Figures S4**. As shown in **Table S2**, all alcohols form smaller clusters on H-MFI compared to H-BEA due to its more confined pore system. The cluster sizes of 1-alcohols (methanol, ethanol, 1-propanol and 1-butanol) are comparable, and its radius is around 4.6 Å on H-MFI and 4.7 Å on H-BEA (the radius is calculated considering the sorbed alcohol molecules form spherical clusters with their

liquid phase densities). Isomeric alcohols such as 2-propanol and 2-butanol/*iso*-butanol form larger clusters compared to 1-propanol and 1-butanol.

Table S2. Estimation of the number of alcohol molecules involved in the cluster formed at the BAS and the correlated volume and radius of the cluster determined from Figure 3 and Figure S4.

Adsorbate	Number of molecules involved in a cluster		$V_{Cluster}$ (Å^3)		$r_{Cluster}$ (Å)	
	H-MFI	H-BEA	H-MFI	H-BEA	H-MFI	H-BEA
Methanol	6.0	4.2	403	282	4.6	4.1
Ethanol	4.2	4.5	407	436	4.6	4.7
1-Propanol	3.3	4.0	409	496	4.6	4.9
2-Propanol	4.2	5.0	533	634	5.0	5.3
1-Butanol	2.0	2.8	304	425	4.2	4.7
2-Butanol	4.7	5.2	715	791	5.5	5.7
iso-Butanol	4.0	4.5	612	689	5.3	5.5

Supplementary Note 4. Additional figures and tables

Table S3. Physicochemical properties of zeolite samples.

Sample	Si/Al	BET surface area $m^2 \cdot g^{-1}$	Micropore volume $cm^3 \cdot g^{-1}$	BAS concentration $mmol \cdot g^{-1}$
H-MFI	45	365	0.12	0.36
Si-MFI	-	334	0.16	-
H-BEA	50	566	0.20	0.21
Si-BEA	-	568	0.19	-

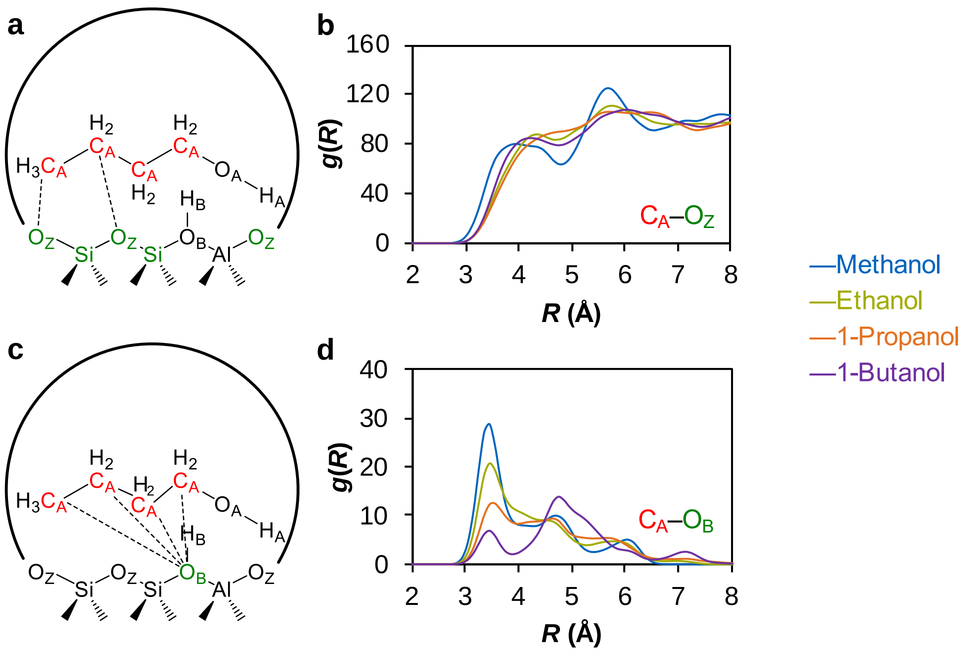

Figure S6. Radial distribution function of C_1–C_4 primary alcohols in H-MFI. (a)–(b) Radial distribution function, $g(R)$, for the alcohol carbon, C_A, to the framework oxygen, O_z, and the BAS oxygen, O_B, (c)–(d) $g(R)$ for C_A to O_B.

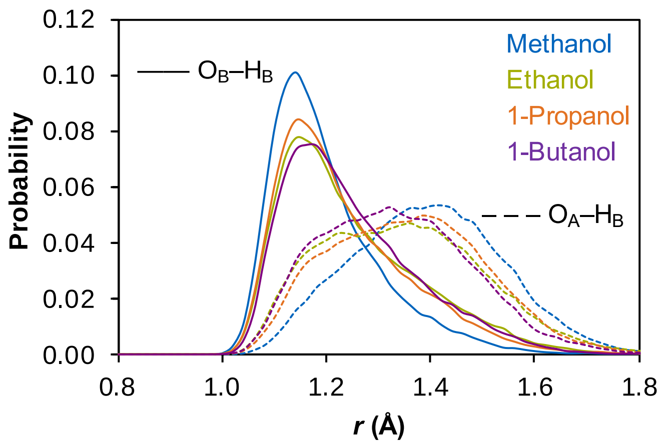

Figure S7. Bond lengths of O_B–H_B (solid lines) and O_A–H_B (dashed lines) for C_1–C_4 primary alcohols in H-MFI. Plotted is a histogram of the probability distribution of the O_B–H_B and O_A–H_B bond lengths. The labels O_B and H_B refer to the BAS oxygen and BAS proton, respectively. O_A refers to the oxygen of the alcohol. Results are based on 240 ps of well-equilibrated AIMD trajectories.

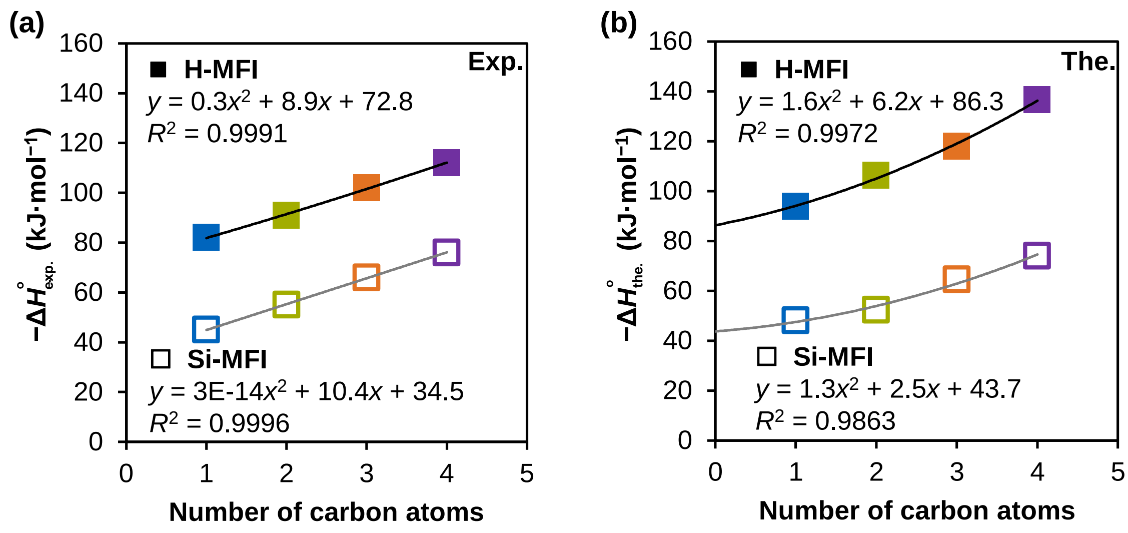

Figure S8. Polynomial fits of the differential heat of adsorption of C_1–C_4 primary alcohols as a function of the number of carbon atoms. Polynomial fits of the differential heat of adsorption of C_1–C_4 primary alcohols as a function of the number of carbon atoms on Si-MFI (open square symbols) and H-MFI (solid square symbols) at 323 K from (a) experimental and (b) computational calculations.

REFERENCES

(1) Wang, M.; Jaegers, N. R.; Lee, M.-S.; Wan, C.; Hu, J. Z.; Shi, H.; Mei, D.; Burton, S. D.; Camaioni, D. M.; Gutiérrez, O. Y.; et al. Genesis and Stability of Hydronium Ions in Zeolite Channels. *Journal of the American Chemical Society* **2019**, *141* (8), 3444-3455. DOI: 10.1021/jacs.8b07969.

(2) Hunter, E. P.; Lias, S. G. Evaluated gas phase basicities and proton affinities of molecules: an update. *Journal of Physical and Chemical Reference Data* **1998**, *27* (3), 413-656.

(3) Osborne, N. S.; Stimson, H. F.; Ginnings, D. C. Measurements of heat capacity and heat of vaporization of water in the range 0 to 100 C. *Precision Measurement and Calibration: Heat and mechanics* **1961**, *77*, 277.

(4) Silverstein, T. P.; Heller, S. T. p K a Values in the Undergraduate Curriculum: What Is the Real p K a of Water? *Journal of Chemical Education* **2017**, *94* (6), 690-695.

(5) Bordwell, F. G. Equilibrium acidities in dimethyl sulfoxide solution. *Accounts of Chemical Research* **1988**, *21* (12), 456-463.

(6) Ugur, I.; Marion, A.; Parant, S.; Jensen, J. H.; Monard, G. Rationalization of the pKa Values of Alcohols and Thiols Using Atomic Charge Descriptors and Its Application to the Prediction of Amino Acid pKa's. *Journal of Chemical Information and Modeling* **2014**, *54* (8), 2200-2213. DOI: 10.1021/ci500079w.
(7) Takahashi, S.; Cohen, L. A.; Miller, H. K.; Peake, E. G. Calculation of the pKa values of alcohols from. sigma. constants and from the carbonyl frequencies of their esters. *The Journal of Organic Chemistry* **1971**, *36* (9), 1205-1209.
(8) Murto, J.; Sandström, J.; Toplin, I.; Melera, A.; Nilsson, L. Nucleophilic Reactivity of Alkoxide Ions Towards 2, 4-Dinitrofluorobenzene and the Acidity of Alcohols. *Acta Chemica Scandinavica* **1964**, *18*, 1043-1053.
(9) P.J. Linstrom and W.G. Mallard, E. *NIST Chemistry WebBook,*; DOI: https://doi.org/10.18434/T4D303,.
